# Antiviral activity of digoxin and ouabain against SARS-CoV-2 infection and its implication for COVID-19

**DOI:** 10.1038/s41598-020-72879-7

**Published:** 2020-10-01

**Authors:** Junhyung Cho, Young Jae Lee, Je Hyoung Kim, Sang il Kim, Sung Soon Kim, Byeong-Sun Choi, Jang-Hoon Choi

**Affiliations:** 1 Division of Viral Disease Research, Center for Infectious Diseases Research, Korea National Institute of Health, Korea Centers for Disease Control and Prevention, 187 Osongsaengmyeong 2-ro, Osong-eup, Heungdeok-gu, Cheongju-si, 28159 Chungcheongbuk-do Republic of Korea; 2 Center for Infectious Diseases Research, Korea National Institute of Health, Korea Centers for Disease Control and Prevention, Cheongju, Republic of Korea; 3 Division of Infectious Disease, Seoul St. Mary’s Hospital, College of Medicine, The Catholic University, Seoul, Republic of Korea

**Keywords:** Cell biology, Drug discovery, Immunology, Microbiology, Diseases

## Abstract

The current coronavirus (COVID-19) pandemic is exacerbated by the absence of effective therapeutic agents. Notably, patients with COVID-19 and comorbidities such as hypertension and cardiac diseases have a higher mortality rate. An efficient strategy in response to this issue is repurposing drugs with antiviral activity for therapeutic effect. Digoxin (DIG) and ouabain (OUA) are FDA drugs for heart diseases that have antiviral activity against several coronaviruses. Thus, we aimed to assess antiviral activity of DIG and OUA against SARS-CoV-2 infection. The half-maximal inhibitory concentrations (IC_50_) of DIG and OUA were determined at a nanomolar concentration. Progeny virus titers of single-dose treatment of DIG, OUA and remdesivir were approximately 10^3^-, 10^4^- and 10^3^-fold lower (> 99% inhibition), respectively, than that of non-treated control or chloroquine at 48 h post-infection (hpi). Furthermore, therapeutic treatment with DIG and OUA inhibited over 99% of SARS-CoV-2 replication, leading to viral inhibition at the post entry stage of the viral life cycle. Collectively, these results suggest that DIG and OUA may be an alternative treatment for COVID-19, with potential additional therapeutic effects for patients with cardiovascular disease.

## Introduction

Human coronaviruses (HCoV) are enveloped, positive-sense single-stranded RNA viruses. Six coronaviruses, namely HCoV-229E, HCoV-OC43, HCoV-HKU1, HCoV-NL63, severe acute respiratory syndrome coronavirus (SARS-HCoV) and Middle East respiratory syndrome coronavirus (MERS-HCoV) are responsible for respiratory illnesses in human. Of these, SARS-CoV and MERS-CoV infection results in severe acute respiratory disease and have caused worldwide awareness of these illnesses^1^.

In December 2019, a novel coronavirus was identified in a group of patients with pneumonia in Wuhan, Hubei province, China^2^. Subsequently, the International Committee on Taxonomy of Viruses (ICTV) named this virus severe acute respiratory syndrome coronavirus 2 (SARS-CoV-2), which is the causative agent of coronavirus disease 19 (COVID-19)^3^. Owing to the substantial increase in the number of confirmed cases of COVID-19 through human-to-human transmission, a large epidemic occurred in Wuhan^4^. As SARS-CoV-2 spread to more than 216 countries, the World Health Organization (WHO) declared an ongoing pandemic on March 11 2020^5^. As of 5 June 2020, more than 6 million confirmed cases and 387,298 deaths have been reported worldwide^6^.

The common symptoms of patients with COVID-19 are mild and most are asymptomatic^7–10^. However, some cases have severe pneumonia, acute cardiac injury, multi-organ failure and death^4,10–12^. Notably, patients with COVID-19 and comorbidities such as hypertension and cardiac diseases show a higher mortality rate than patients without cardiac diseases (51.2% vs. 5.1%)^7,8,11,13,14^.

No approved vaccine or specific antiviral agent is yet available for COVID-19. One of the fastest and most practical strategies to address this issue is to identify preexisting approved drugs with antiviral activity against SARS-CoV-2. Accordingly, numerous clinical trials and studies are underway to evaluate vaccines and therapeutics, with the most well-studied clinical trial-repurposed drugs being (hydroxy)chloroquine and remdesivir (REM), the latter of which received emergency use authorization by the U.S. Food and Drug Administration (FDA) on May 1, 2020^15,16^. Moreover, the WHO announced a large global clinical trial, SOLIDARITY, to assess the therapeutic effects of four repurposed drugs on March 18, 2020.

In addition to antiviral drugs based on viral protease inhibitors and nucleoside analogs, the cardiac glycoside (CG)-based drugs digoxin (DIG) and ouabain (OUA) have been shown to exhibit antiviral activity through various mechanisms against several DNA and RNA viruses, such as cytomegalovirus, herpes simplex virus, MERS-CoV, human immunodeficiency virus, respiratory syncytial virus, chikungunya virus, and the recently identified SARS-CoV-2^17–24^.

Notably, these agents have been used to treat various heart diseases and were mainly identified to bind to the transmembrane protein sodium/potassium ATPase (Na^+^/K^+^-ATPase) and inhibit ion-exchange, leading to increased intracellular Ca^++^ concentration and heart muscle contraction^25–27^. Therefore, in this study, we evaluated the antiviral activity of DIG and OUA based on viral growth kinetics and inhibition at different stages of viral infection, and compared it to that of vehicle (DMSO), chloroquine (CHQ) and REM to identify a suitable and potent antiviral agent to treat COVID-19 patients with cardiac diseases.

## Results

### Determination of half maximal inhibitory, cell cytotoxic concentration and selective index value of DIG and OUA

**Figure 1:**
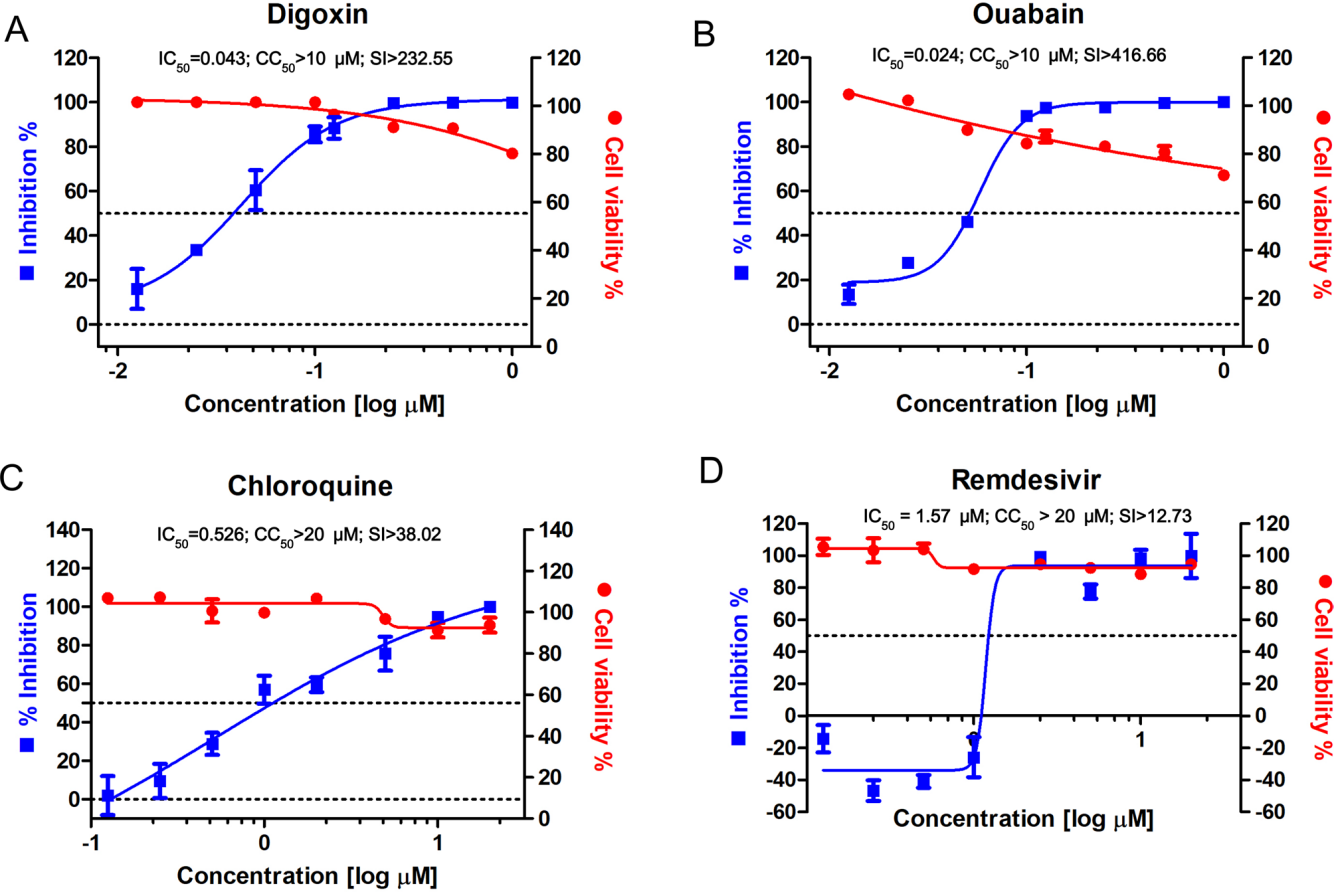
Half maximal inhibitory concentration (IC_50_)_,_ cytotoxic concentration (CC_50_) and selective index (SI) of digoxin, ouabain, chloroquine and remdesivir against SARS- CoV-2 infection. IC_50_ (left axis, blue square), CC_50_ (right axis, red circle), and SI of **(A)** digoxin (DIG), **(B)** ouabain (OUA), **(C)** chloroquine (CHQ) and **(D)** remdesivir (REM). Vero cells were infected with BetaCoV/Korea/KCDC03/2020 at an MOI of 0.01 in the presence of the indicated drug concentration for 1 h. Subsequently, the cells were washed and incubated in the presence of the indicated drug concentration for 24 h. IC_50_ and CC_50_ were determined from dose response curves based on treatment with eight concentrations. IC_50_ were determined by viral copy number based on standard curve, and CC_50_ was investigated by cell viability assay. Viral copy number in DMSO was set to 100%, and the remaining values as means ± SD (n = 3). Corresponding viral mRNA expression levels are shown in Supplementary Figure S1.

To assess the antiviral activity and cell cytotoxicity of DIG and OUA against SARS-CoV-2 infection, we investigated their half-maximal inhibitory concentration (IC_50_), cytotoxicity concentration 50% (CC_50_) and selective index (SI), and compared to those of CHQ. Vero cells were infected with BetaCoV/Korea/KCDC03/2020 at a multiplicity of infection (MOI) of 0.01 in the presence of OUA and DIG (0.0125, 0.025, 0.05, 0.1, 0.125, 0.25, 0.5 and 1 μM), CHQ (0.125, 0.25, 0.5, 1, 2, 5, 10 and 20 μM) and REM (0.125, 0.25, 0.5, 1, 2.5, 5, 10 and 20 μM) for 1 h and incubated with the respective drug for 24 h. The viral copy numbers in the cell culture supernatant were determined by amplifying the nucleocapsid (*N*) gene by quantitative real-time PCR (qRT-PCR), and cell viability was measured using the PrestoBlue Cell Viability reagent. The IC_50_ values of DIG (IC_50_ = 0.043 μM) and OUA (IC_50_ = 0.024 μM) were determined at a nanomolar concentration, and were over tenfold lower than those of CHQ (IC_50_ = 0.526 μM), and REM (IC_50_ = 1.57 μM) (Fig. 1A–D). In addition, the SI (CC_50_/IC_50_) values of DIG (CC_50_ > 10 μM, SI > 232.55) and OUA (CC_50_ > 10 μM, SI > 416.66) were over fivefold greater than those of either CHQ (CC_50_ > 20 μM, SI > 38.02) or REM (CC_50_ > 20 μM, SI > 12.73) (Fig. 1A–D).

### Antiviral activity of DIG and OUA based on SARS-CoV-2 growth kinetics

To evaluate antiviral activity of drugs based on SARS-CoV-2 growth kinetics, cells were treated with optimal concentrations of DIG (150 nM), OUA (100 nM), CHQ (10 μM) and REM (10 μM), which inhibited over 95% of viral *N* mRNA expression at 24 hpi (Supplementary Fig. S1A–E).

**Figure 2:**
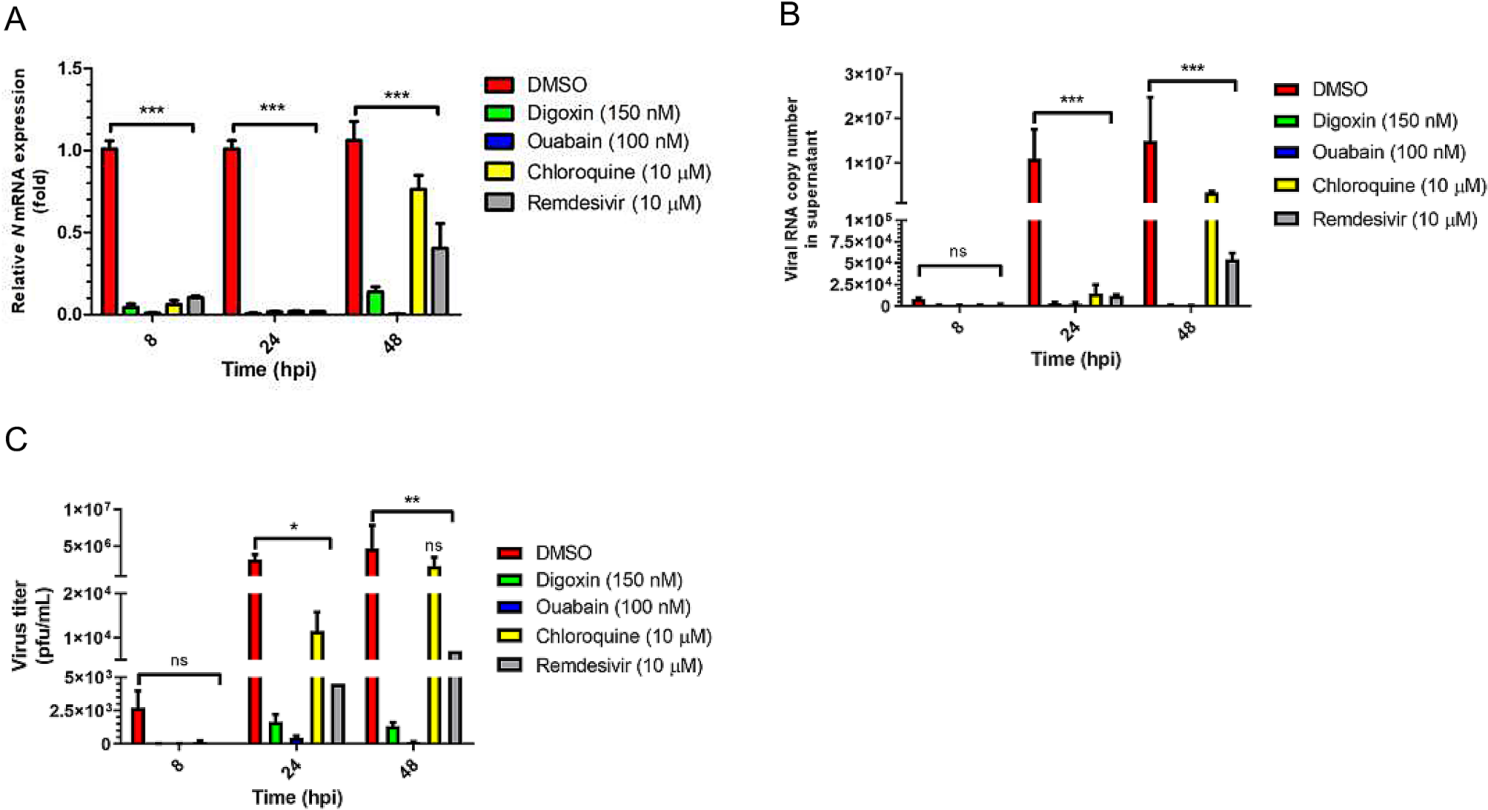
Antiviral activity of digoxin, ouabain, chloroquine and remdesivir on SARS-CoV-2 growth kinetics. Vero cells were infected with BetaCoV/Korea/KCDC03/2020 at an MOI of 0.01 in the presence of DIG (150 nM), OUA (100 nM), CHQ (10 μM) and REM (10 μM) for 1 h. Subsequently, the cells were washed and incubated in the presence of the indicated drug for 8, 24, and 48 hpi. At each time point, **(A)** viral mRNA expression, **(B)** viral copy number and **(C)** progeny titer were assessed using qRT-PCR and plaque assays. Viral mRNA expression was normalized to *GAPDH* expression. DMSO was set to 1, and the remaining values are represented as a relative value. Viral copy number was calculated using a standard curve. Values are presented as mean ± SD (n = 3). Statistically significantly differences between DMSO and drug treatment are represented as *P < 0.05, **P < 0.01 and ***P < 0.001 determined using the two-way ANOVA with Bonferroni post-tests (each column compared to control). *ns* not significant.

After treating the virus-infected cells with drugs, viral *N* mRNA expression, viral copy number, and progeny virus titer were measured at 8, 24 and 48 h (the duration of the complete virus replication was assessed via growth kinetics) (Fig. 2A–C). Viral *N* mRNA expression was almost fully suppressed (> 99%) by all drugs at 8 and 24 hpi. However, the expression of viral *N* mRNA was significantly restored in CHQ-treated cells at 48 hpi (Fig. 2A). Viral copy numbers were also markedly reduced at 48 h in the DIG-, OUA-, and REM-treated cell culture supernatants (Fig. 2B). Progeny virus titer in the culture medium of DIG, OUA and REM treatment, as measured using plaque assays, revealed a 10^3^- and 10^4^-fold reduction, respectively, and an inhibition ratio of > 99% for the drugs compared to those for the carrier (DMSO, 1.80 × 10^6^ plaque forming units (pfu)/mL). Moreover, the virus titer was considerably reduced in the cells treated with DIG (1.83 × 10^3^ pfu/mL), OUA (1.80 × 10^2^ pfu/mL) and REM (7.0 × 10^3^ pfu/mL), but not CHQ (2.45 × 10^6^ pfu/mL) at 48 hpi (Fig. 2C, Table 1 and Supplementary Fig. S2).

**Table 1:** Progeny virus titer in cell supernatant.

Time (hpi)	Drug	Pfu/mL
8	DMSO	3.07 × 10^3^
	Digoxin	1.67 × 10^1^
	Ouabain	1.67 × 10^1^
	Chloroquine	1.60 × 10^2^
	Remdesivir	0.66 × 10^1^
24	DMSO	3.90 × 10^6^
	Digoxin	1.43 × 10^3^
	Ouabain	6.53 × 10^2^
	Chloroquine	1.37 × 10^4^
	Remdesivir	4.50 × 10^3^
48	DMSO	1.80 × 10^6^
	Digoxin	1.83 × 10^3^
	Ouabain	1.80 × 10^2^
	Chloroquine	2.45 × 10^6^
	Remdesivir	7.00 × 10^3^

### Determination of the inhibition step in the SARS-CoV-2 life cycle by drug treatment

**Figure 3:**
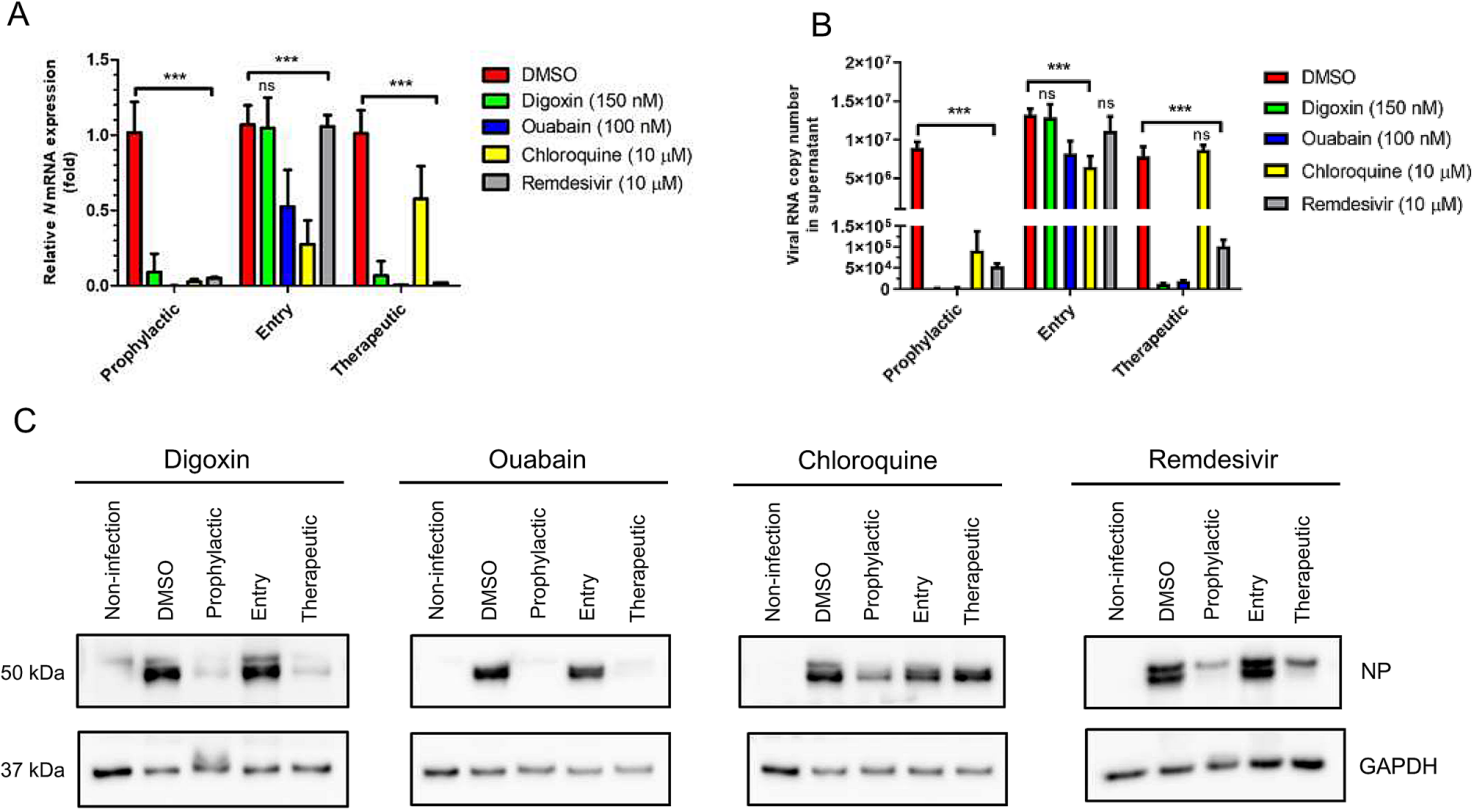
Determination of the inhibition step of each drug in the SARS-CoV-2 life cycle. Vero cells were infected with BetaCoV/Korea/KCDC03/2020 at an MOI of 0.01 and treated with DIG (150 nM), OUA (100 nM), CHQ (10 μM) and REM (10 μM) in prophylactic, entry, and therapeutic conditions. Then, the cells were incubated with the indicated drug or without drug in fresh media for 24 h. Viral **(A)** mRNA expression, **(B)** copy number, and **(C)** N protein expression were investigated using qRT-PCR and western blotting (also see Supplementary Figure S3; full image of the blots). Viral mRNA expression was normalized to *GAPDH* levels and represented as relative values. Values are presented as mean ± SD (n = 3). Anti-GAPDH blots were used as loading controls. Viral NP protein and GAPDH protein were blotted in the same gel. Statistically significantly differences between DMSO and drug treatment are represented as **P < 0.01 and ***P < 0.001 determined using the two-way ANOVA with Bonferroni post-tests. *ns* not significant.

To determine which step of the virus life-cycle is inhibited by drug treatment, DIG, OUA, CHQ and REM were administered at the different time points of treatment: prophylactic (1 h prior to infection and maintenance for 24 h), entry (0 h of infection and maintenance for 2 h), and therapeutic (2 h following infection and maintenance for 24 h). All drugs demonstrated high efficacy upon prophylactic administration. Viral copy number, mRNA expression, and viral N protein expression were lower in prophylactic-treated cells than in non-treated cells by approximately 99% (Fig. 3). In entry-treated cells, CHQ and OUA treatment significantly inhibited viral RNA and protein levels to approximately 60% and 30% of those of DMSO, respectively, whereas DIG and REM treatment did not effectively inhibit virus propagation (Fig. 3A–C). In comparison, OUA, DIG, and REM, but not CHQ, treatment markedly reduced viral replication in the therapeutic-treated cells (Fig. 3A–C).

## Discussion

SARS-CoV-2 infection causes not only multiple organ failure but also higher mortality rate in patients with underlying cardiac diseases^4,28,29^. Notably, the angiotensin-converting enzyme 2 (ACE2) receptor, which serves as a functional receptor for coronaviruses, is systemically distributed in multiple organs and is especially highly expressed in the heart and lungs^29^. Therefore, these organs may be directly attacked by SARS-CoV-2 or indirectly damaged by elevated levels of proinflammatory cytokines^30–35^.

The drugs DIG and OUA have been used to treat heart conditions of patients for over 10 decades, and thus their clinical dosage regimen, bioavailability, pharmacokinetic profile information, and safety are well known^27^. Hence, these drugs may exert multiple benefits in patients with COVID-19 in terms of antiviral and symptom management and safety. Although the IC_50_ in an in vitro study is a poor guide for clinically relevant concentrations of DIG and OUA, previous studies of cancer therapy with DIG using human cells reported IC_50_ ranges from 0.02 to 0.34 μM, and the correspond plasma concentrations were safe and in the acceptable range from 0.8 to 2.6 nM (1 ng/mL = 1.28 nmol/L) in patients with cardiac diseases^27,39^. Moreover, the serum levels of patients taking oral doses of 0.25 mg/day (3.4 to 5.1 μg/kg/day) DIG are in the range from 1 to 2.6 nM^40,41^. Therefore, the IC_50_ of DIG and OUA described in this study may be helpful for further pre-clinical and clinical studies.

In this study, single dose of DIG and OUA treatment consistently showed superior antiviral activity against human isolate BetaCoV/Korea/KCDC03/2020 infection, as evident from the evaluation of viral mRNA expression, copy number (released virions in cell supernatant), and progeny virus titer up to 48 hpi in vitro. The progeny virus titer at 48 hpi in the DIG and OUA treatment groups was comparable to that in REM and reduced more than 10^3^–10^4^-fold compared to either the non-treated vehicle (DMSO) or CHQ groups, indicating that DIG and OUA have effective antiviral activity with stability up to 48 hpi (this time point represents the peak viral titer on growth kinetics in Vero cells, corresponding to maximal SARS-CoV-2 replication)^36^. Moreover, DIG and OUA treatment significantly inhibited over 99% of viral mRNA expression, which is more effective than REM (> 60%) and CHQ (> 30%) at 48 hpi. These results suggest that DIG and OUA could be an alternative treatment against SARS-CoV-2 infection.

Notably, DIG and OUA significantly inhibited viral mRNA expression, copy number, and viral protein expression when administered at the post-entry stage, although DIG did not show effective antiviral activity at the host entry stage of the virus cycle. Clinically, these results are very important for therapeutics, as a large numbers of patients are asymptomatic at the initial stage of SARS-CoV-2 infection^37,38^. Moreover, the results indicate that the inhibition mechanism of SARS-CoV-2 by DIG may be similar to that of respiratory syncytial virus (RSV), wherein inhibition occurs at the step of viral RNA synthesis^18^. However, OUA may have another inhibition mechanism, as OUA treatment at the entry stage inhibited approximately 30% of viral mRNA and protein expression. This suggests that an OUA may have an alternative antiviral mechanism related to blocking Src-mediated endocytosis in the entry step of coronaviruses^42^. Interestingly, a recent study reported that digitoxin, a CG, suppresses proinflammatory cytokines in influenza A virus-infected cotton rat lung^43^, which suggests that DIG and OUA may have an additional therapeutic role against COVID-19 with hypercytokinemia.

Taken together, we demonstrated a more effective antiviral activity of DIG and OUA against SARS-CoV-2 infection in vitro than previously approved antiviral agents such as chloroquine and remdesivir. We propose that these agents may be used as therapeutic options for patients with COVID-19 and comorbid cardiac diseases.

## Materials and methods

### Virus, cells, compounds, and infection

BetaCoV/Korea/KCDC03/2020 (GISSAID accession ID: EPI_ISL_407193) was obtained from the National Culture Collection for Pathogens of Korea Center for Disease Control and prevention (KCDC)^44^. Vero cells were purchased from the American Type Culture Collection (ATCC CCL-81, Manassas, VA, USA) and cultured in Dulbecco’s minimal essential medium (DMEM; Gibco, Grand Island, NY, USA) supplemented with 2 (infection media) or 10% (growth media) v/v heat inactivated fetal bovine serum (FBS; Gibco) and 1% v/v penicillin/streptomycin (p/s; Gibco) in a humidified incubator of 5% CO_2_ atmosphere at 37 °C. OUA, CHQ and DIG were purchased from Sigma-Aldrich (St. Louis, MO, USA). REM was purchased from MedChemExpress (Monmouth Junction, NJ, USA). DIG tablets (inno.N, Seoul, Korea) were provided by Dr. Sang il Kim. OUA and DIG tablets were dissolved in H_2_O and CHQ, DIG and REM were dissolved in DMSO. Confluent Vero cells were infected at an MOI of 0.01 of BetaCoV/Korea/KCDC03/2020 in DMEM containing 2% FBS and 1% p/s for 1 h. Following incubation, cells were washed twice with phosphate buffered saline (PBS; Gibco) and incubated in the infection media. All experiments were performed in biosafety level 3 facilities according to KCDC guidelines.

### Cloning and linearity determination of the RNA reference

The PCR products with SARS-CoV-2 N primers were cloned into pGEM-T Easy Vector (Promega, Madison, WI, USA) and in vitro transcribed using the RiboMAX Large Scale RNA Production System-T7 (Promega). The linearity of the RNA reference template was evaluated with a tenfold serial dilution of in vitro transcribed *N* RNA of SARS-CoV-2 (10^4^–10^10^ copies). The copy number of RNA was calculated using: [X g/μL RNA/(transcript length in nucleotides – 340)] × 6.022 × 10^23^ = Y molecules/μL^45^. The linear range was determined using a standard curve generated with diluted reference RNAs and the best-fit line to the raw data was established by linear regression analysis with 95% confidence intervals using GraphPad Prism (version 5.01; La Jolla, CA, USA).

### Quantitative real-time PCR (qRT-PCR)

For the quantification of viral copy number, total RNA was isolated from cell supernatants using the QIAamp viral RNA mini kit (Qiagen, Hilden, Germany) and cDNA was synthesized from 1 μg of total RNA using SuperScript IV (Invitrogen, Waltham, MA, USA) according to the manufacturer’s protocol. qRT-PCR was performed using Power SYBR Green PCR master mix (Applied Biosystems, Foster City, CA, USA) and an Applied Biosystems QuantStudio3 (Applied Biosystems) following the manufacturer’s protocol.

For measurement of viral mRNA, total RNA was isolated using TRIzol (Ambion, Leicestershire, UK) reagent. Subsequently, cDNA was synthesized from 1 μg of total RNA using a SuperScript IV (Invitrogen). qRT-PCR was performed using Power SYBR Green PCR master mix (Applied Biosystems) and Applied Biosystems QuantStudio3 (Applied Biosystems) as follows: denaturation at 95 ℃ for 5 min, followed by 40 cycles of 95 ℃ for 30 s and 60 ℃ for 30 s. The sequences of the *GAPDH* primers used were: 5′-GAA CGG GAA GCT TGT CAT CAA TGG-3′ and 5′-TGT GGT CAT GAG TCC TTC CAC GAT-3′. The sequences of the *N* primers used were: 5′-GGG AGC CTT GAA TAC ACC AAA A-3′ and 5′-TGT AGC ACG ATT GCA GCA TTG-3′^46^.

### Western blotting

Vero cells were lysed in Pierce RIPA buffer (Thermo Scientific, Waltham, MA, USA) and 20 μg of protein was separated using Bolt 4–12% Bis–Tris Plus (Invitrogen). Proteins were detected by probing the membranes with 1:1000 anti-SARS-CoV-2 nucleocapsid (Sino Bio., Beijing, China) and 1:2000 anti-GAPDH (Cell Signaling Technologies, Danvers, MA, USA) antibodies. Protein transfer was performed using iBlot 2 (Invitrogen) and iBlot 2 PVDF Regular Stacks (Invitrogen). Membranes were incubated with 1:2000 goat anti-rabbit antibody (Cell Signaling) conjugated with horseradish peroxidase for 1 h. Then, membranes were washed five times with tris-buffered saline with 5% Tween 20. Thereafter, the blots were detected using Super Signal (Thermo Scientific).

### Plaque assay

Monolayers of Vero cells were prepared in 12-well plates. The cells were infected with tenfold serial dilutions of supernatant from treated cells and incubated at 37 °C for 1 h. The medium was removed and cells were washed with PBS. Each well was overlaid with MEM/agarose (Gibco) and maintained at room temperature until the overlay turned solid. The plates were incubated at 37 °C for 3 days. The cells were then fixed with 2% paraformaldehyde (Thermo Scientific) and stained with 1% crystal violet (Sigma-Aldrich) overnight.

### IC_50_ and CC_50_ measurements

Confluent Vero cells in 12-well plates were pre-treated with eight concentrations of OUA and DIG (0.0125, 0.025, 0.05, 0.1, 0.125, 0.25, 0.5 and 1 μM), CHQ (0.125, 0.25, 0.5, 1, 2, 5, 10 and 20 μM) and REM (0.125, 0.25, 0.5, 1, 2.5, 5, 10 and 20 μM) for 1 h in DMEM containing 2% FBS and 1% p/s. After incubation, an MOI of 0.01 of BetaCoV/Korea/KCDC03/2020 was added to cells for 1 h, cells were washed twice with PBS, and a new medium was added with the indicated concentrations of the drugs for 24 h. Subsequently, cell viability was measured using the PrestoBlue Cell Viability reagent (Invitrogen), and viral RNA was isolated from cell supernatants and cDNA was synthesized. qRT-PCR was performed using Power SYBR Green PCR master mix (Applied Biosystems) and Applied Biosystems QuantStudio3. The copy number was calculated based on the reference RNA template and the IC_50_ value was calculated using GraphPad Prism.

### Drug treatment

Vero cells were pre-treated with DIG (150 nM), OUA (100 nM), CHQ (10 μM) and REM (10 μM) for 1 h, and then the virus was applied for 1 h to allow infection. The drug-virus mixture was removed and the cells were washed twice with PBS. Subsequently, the cells were incubated in the presence of fresh medium containing the optimal concentrations of the drugs, and the cells and supernatant were collected at 8, 24 and 48 hpi for quantification of viral mRNA, copy number, and progeny virus titer.

For the prophylactic condition, Vero cells were pre-treated with the drugs in infection medium for 1 h and the virus was applied for 2 h to allow infection. Subsequently, the cells were washed twice with PBS and incubated for 24 h in the presence of the drugs in fresh infection medium.

For the entry condition, the cells were treated with the drugs for the infection period (2 h), followed by removal of the drug-virus mixture and washing of the cells. Subsequently, the cells were incubated in infection medium without the drugs for 24 h.

For the therapeutic condition, following viral infection without the drugs for 2 h, the virus was discarded and the cells were washed. Then, drugs in fresh infection medium were added to the cells for 24 h.

### Statistical analysis

The statistical significance of DMSO control and drug treatments were assessed by one-way ANOVA with Dunnett’s multiple comparison test. The statistical comparison of the viral copy number, mRNA expression, and progeny virus titer was performed using two-way ANOVA with Bonferroni post-tests. Data plotting and statistical analysis were performed using GraphPad Prism. A *P* value < 0.05 was considered statistically significant.

## Supplementary information

Supplementary Information.

## Data Availability

The data that support the findings of this study are available from the corresponding author on reasonable request.
