# Supplementary material for: Antiviral activity of digoxin and ouabain against SARS-CoV-2 infection and its implication for COVID-19
Source: Sci Rep. 2020 Oct 1;10:16200. doi: 10.1038/s41598-020-72879-7 (PMC7530981; doi:10.1038/s41598-020-72879-7)
Supplement: Supplementary file 1 — Supplementary Information. [file 41598_2020_72879_MOESM1_ESM.docx]

**Antiviral Activity of Digoxin and Ouabain against SARS-CoV-2 Infection and Its Implication for COVID-19**

Junhyung Cho,^1^ Young Jae Lee,^1^ Je-Hyoung Kim,^1^ Sang il Kim,^3^ Sung Soon Kim,^2^ Byeong-Sun Choi,^1,*^ and Jang-Hoon Choi^1,*^

^1^Division of Viral Disease Research, Center for Infectious Diseases Research, Korea National Institute of Health, Korea Centers for Disease Control and Prevention, Cheongju, Republic of Korea

^2^Center for Infectious Diseases Research, Korea National Institute of Health, Korea Centers for Disease Control and Prevention, Cheongju, Republic of Korea

^3^Division of Infectious Disease, Seoul St. Mary’s Hospital, College of Medicine, the Catholic University, Seoul, Republic of Korea

Authors

^1^Junhyung Cho (J. C), Ph.D, email: cautionjun@Korea.kr

^1^Young Jae Lee (Y. J. L), Ph.D, email: yjofage@Korea.kr

^1^Je-Hyoung Kim (J. H. K), Ph.D, email: kg0705@Korea.kr

^3^Sang il Kim (S. I. K), MD, Ph.D, email: drksi@catholic.ac.kr

^2^Sung Soon Kim (S. S. K), Ph.D, email: [sungskim63@gmail.com](mailto:sungskim63@gmail.com)

*Byeong-Sun Choi (B. S. C), Ph.D, email: [byeongsun@Korea.kr](mailto:byeongsun@Korea.kr)

*Jang-Hoon Choi (J. H. C), Ph.D, email: jhchoi@nih.go.kr

*Corresponding author at: Division of Viral Disease Research, Center for Infectious Diseases Research, Korea National Institute of Health, Korea Centers for Disease Control and Prevention

187, Osongsaengmyeong2-ro, Osong-eup, Heungdeok-gu, Cheongju-si, Chungcheongbuk-do 28159, Republic of Korea

Phone: +82-43-719-8415

Fax: +82-43-719-8459

Email: jhchoi@nih.go.kr (J.H.C.); byeongsun@Korea.kr (B.S.C.)

Supplementary Figure S1


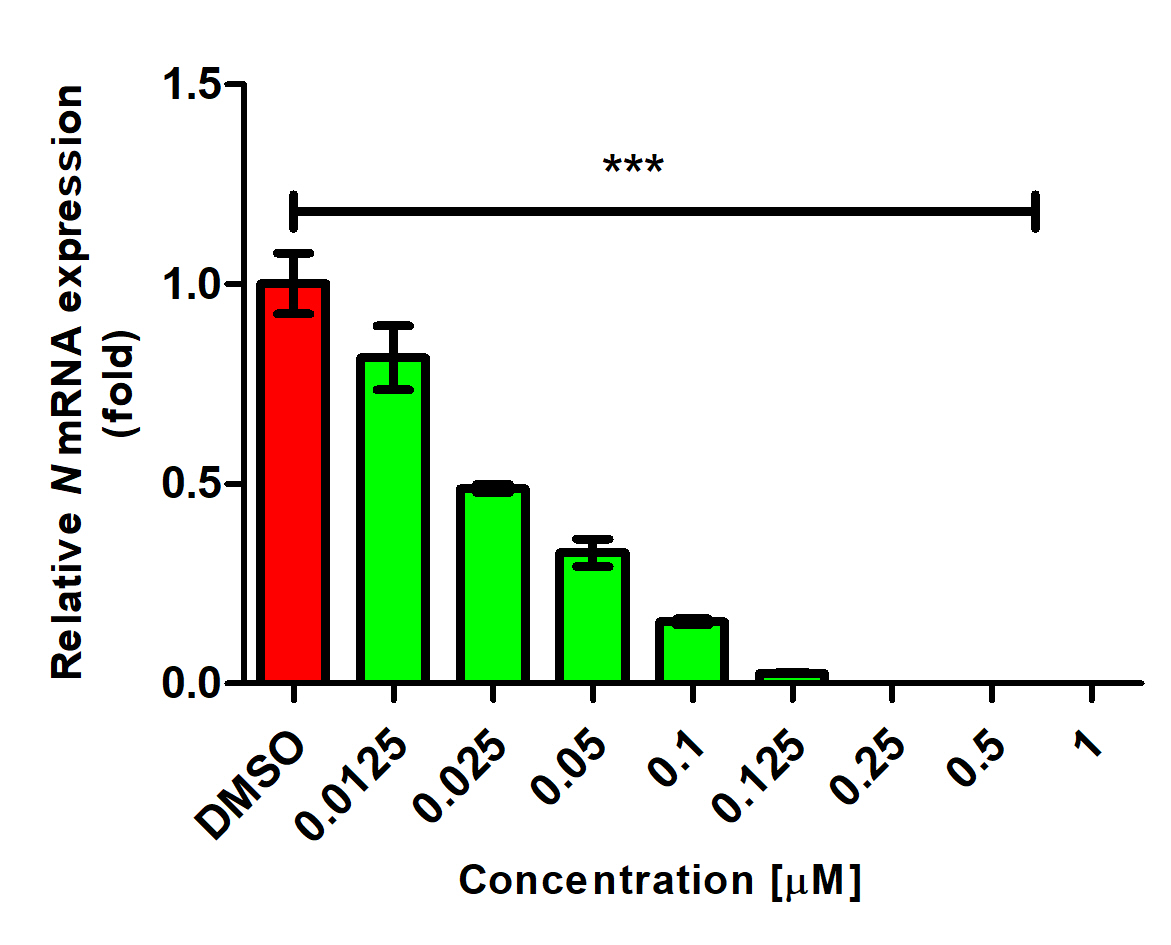


E

D

A

B

C


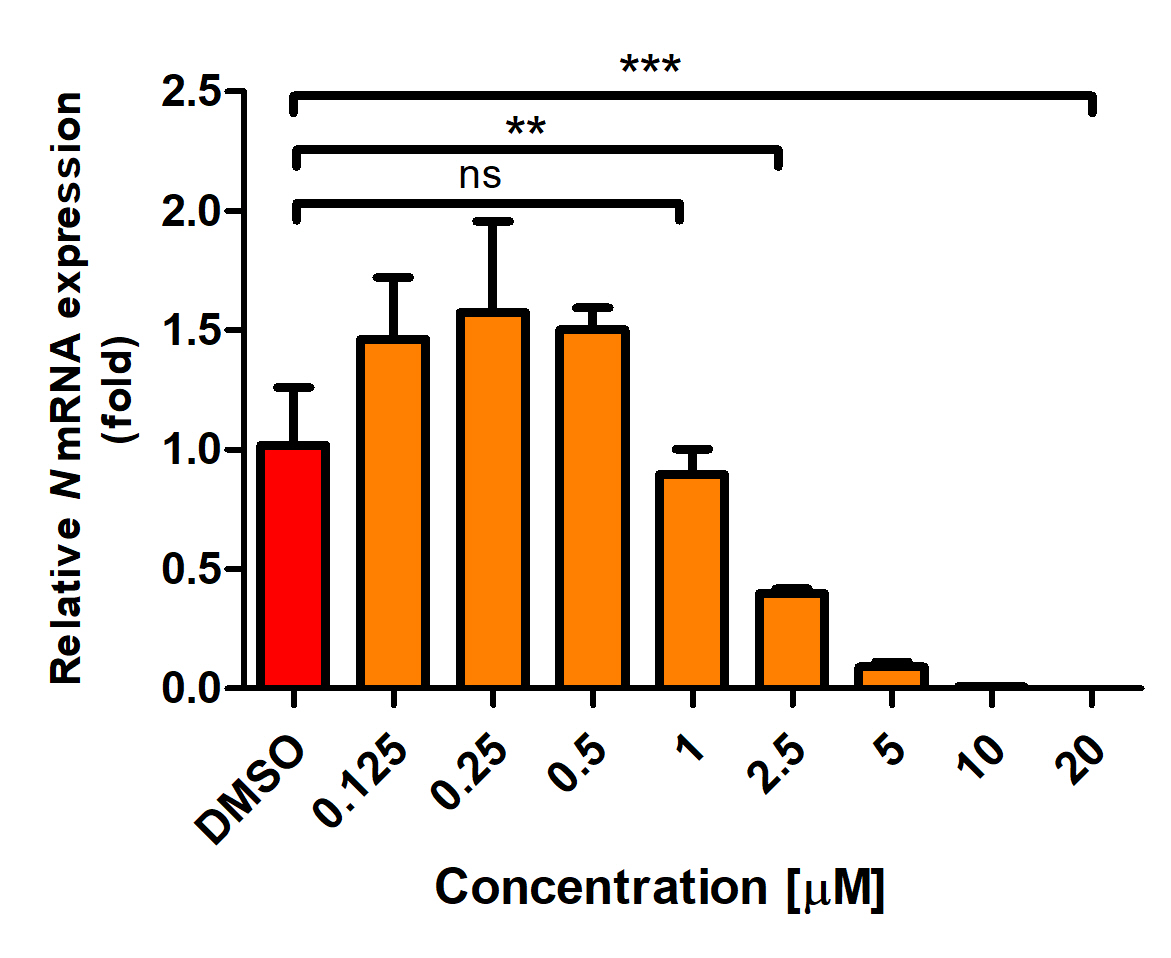

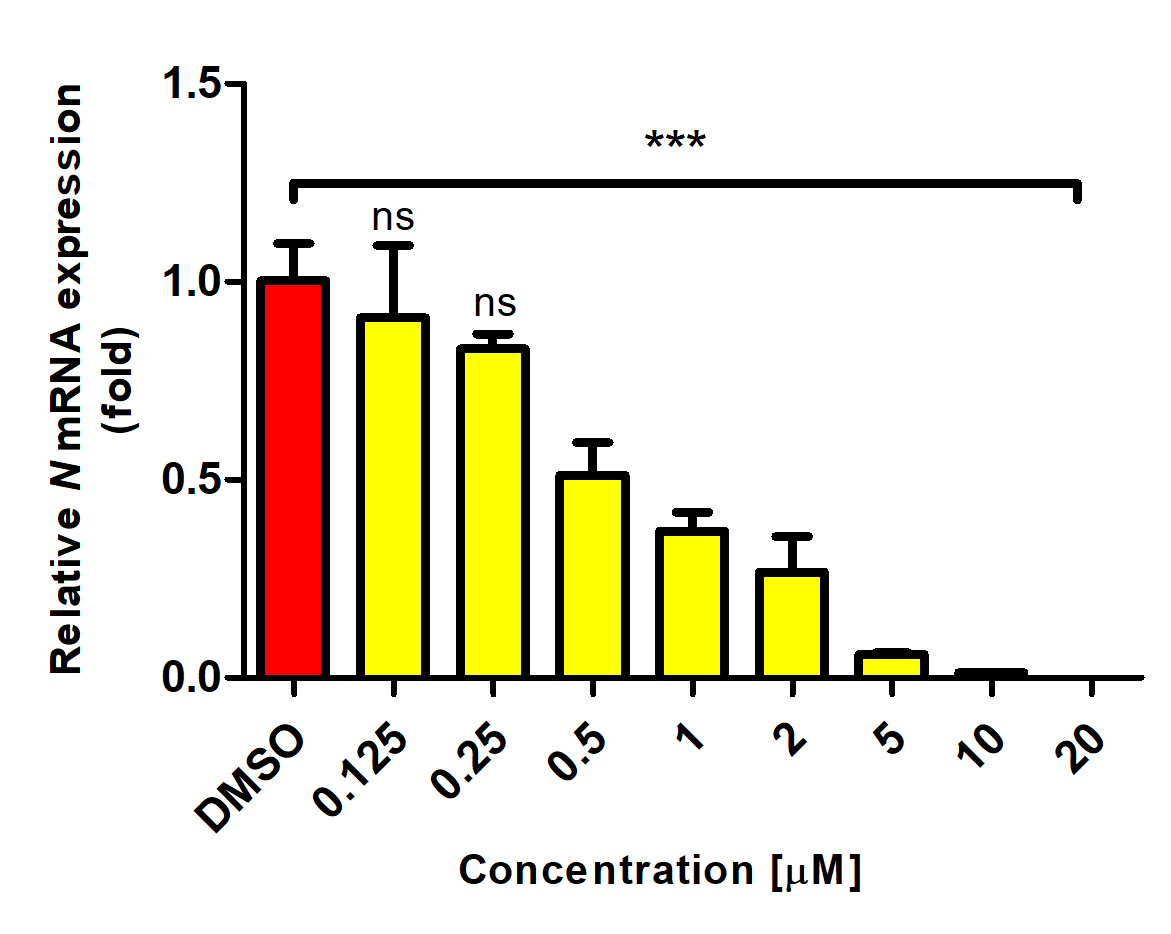

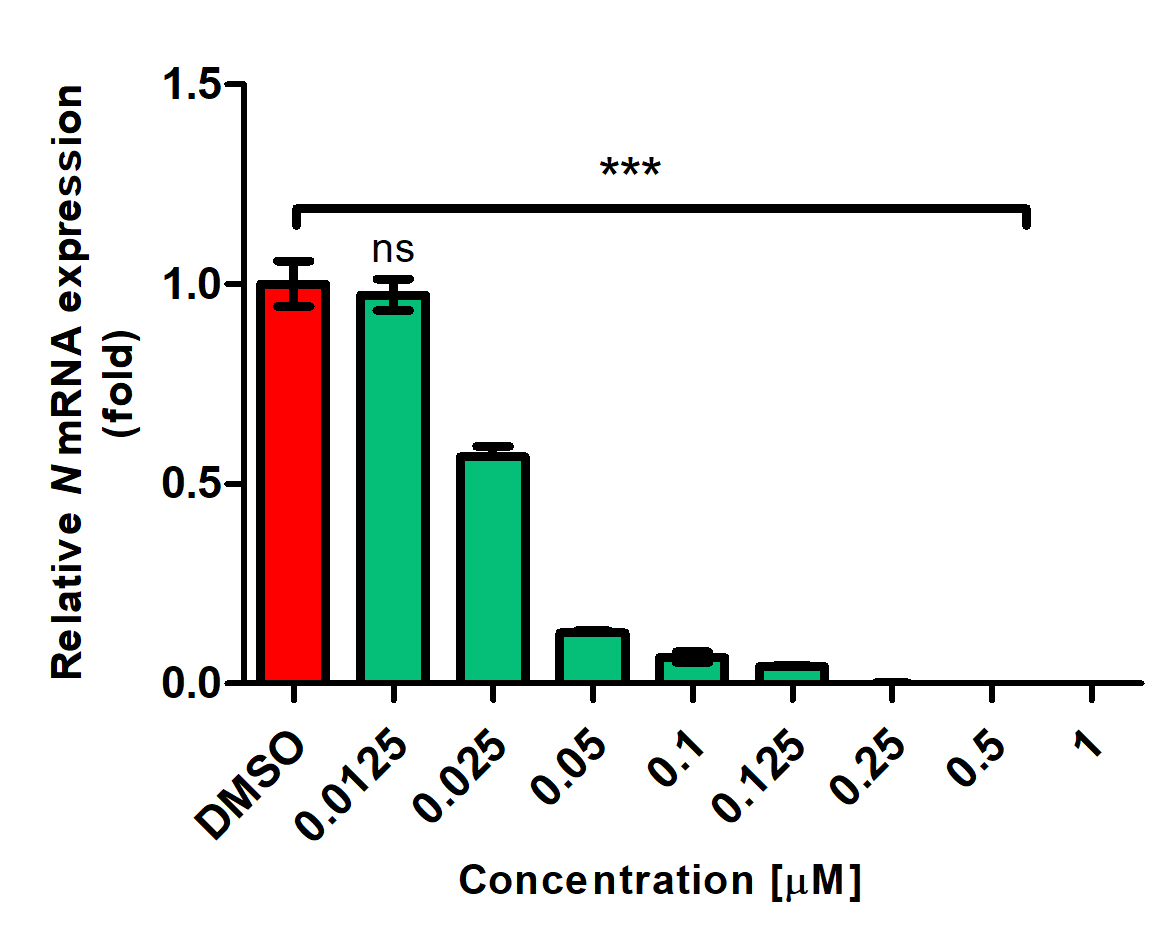

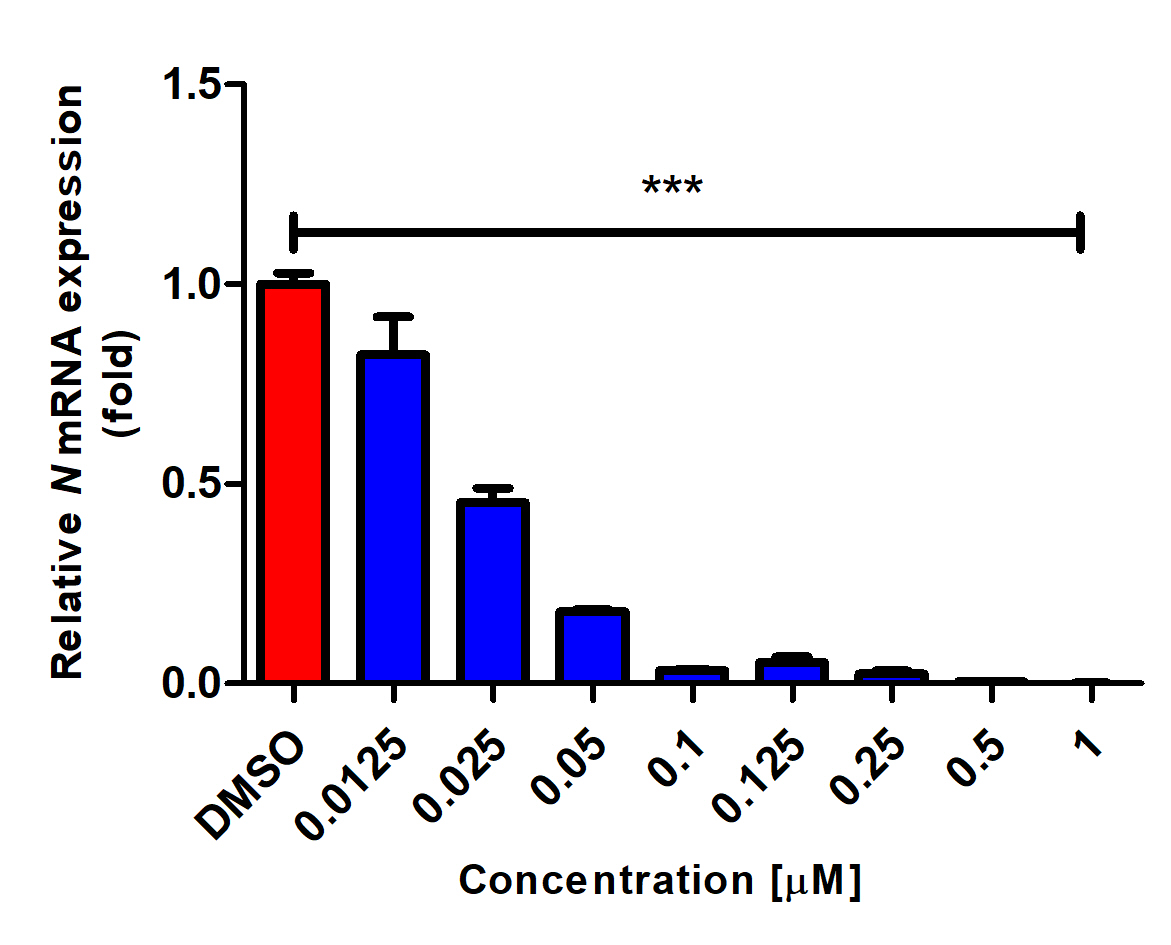


Supplementary Figure S2


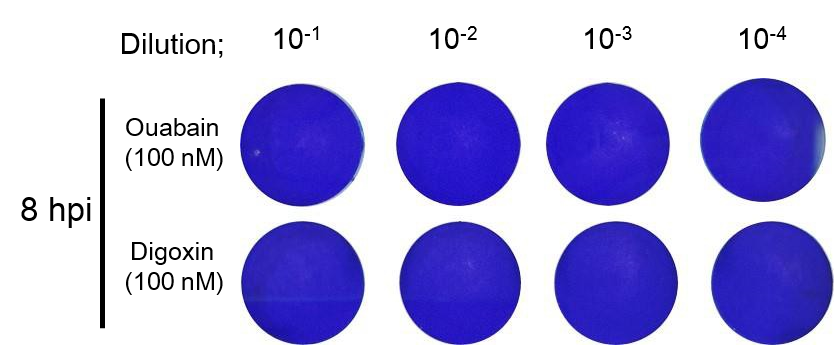


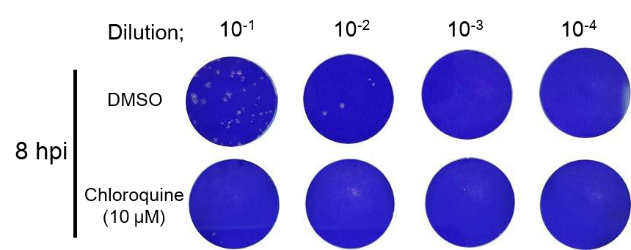


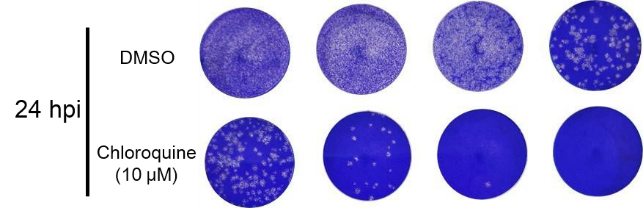


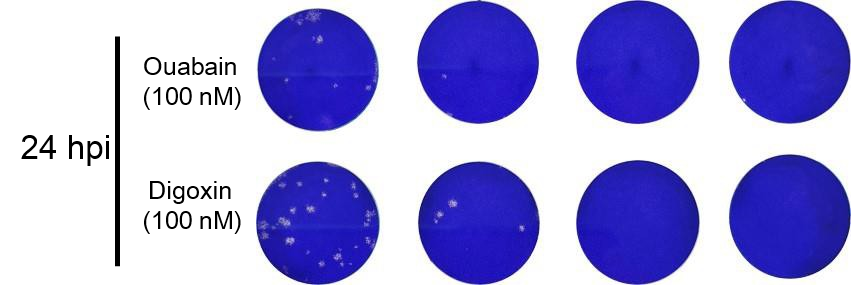

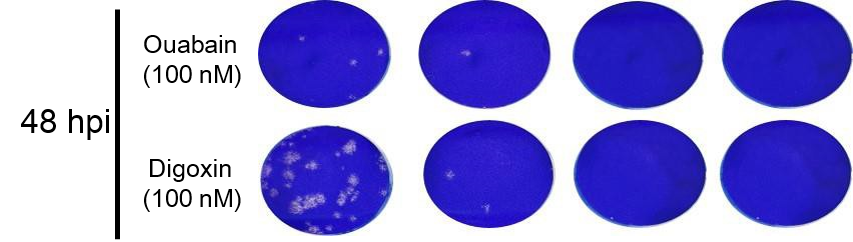

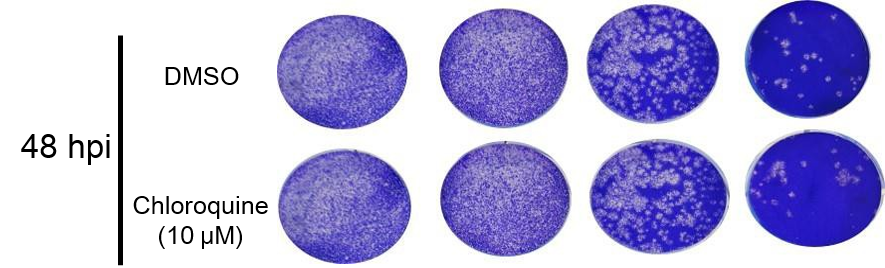

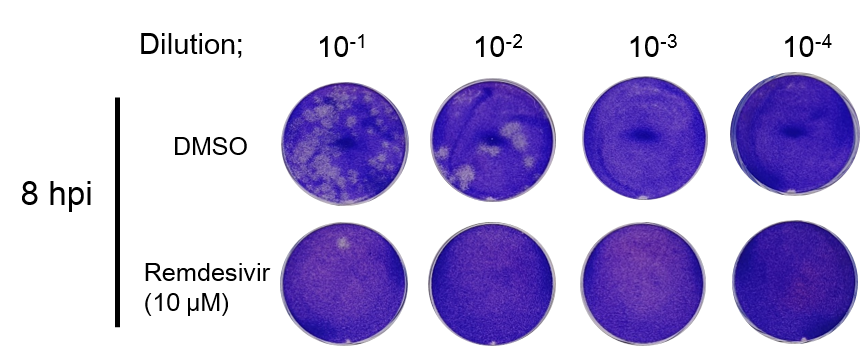

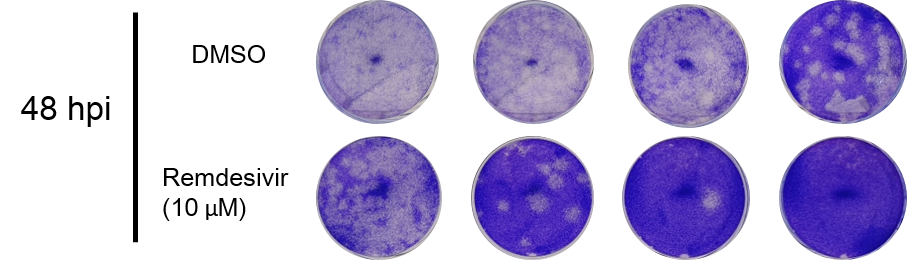

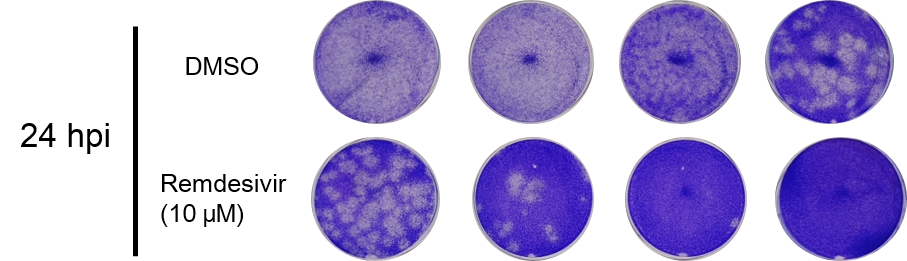


Supplementary Figure S3


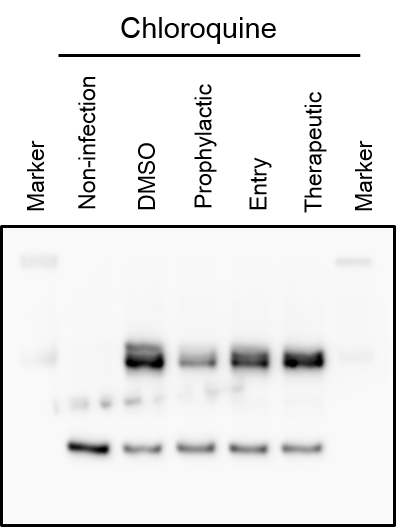

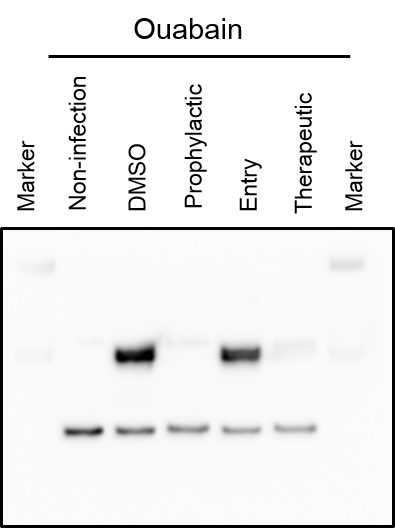

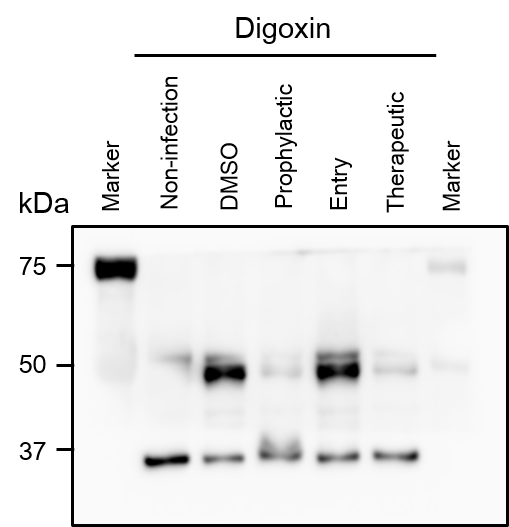

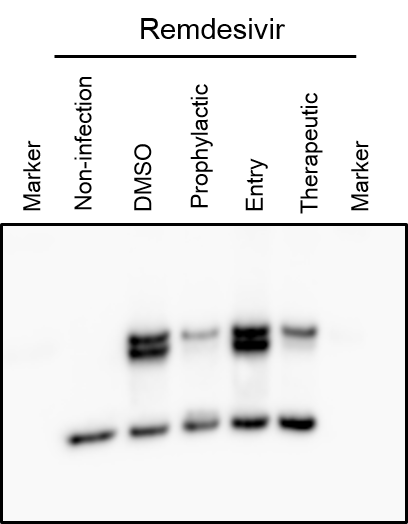


# Supplementary Figure legends

**Figure S1. Drug dose-dependent changes in SARS-CoV-2 mRNA expression**

Vero cells were infected with BetaCoV/Korea/KCDC03/2020 at a multiplicity of infection of 0.01 in the presence of designated concentrations of (A) digoxin, (B) digoxin tablets, (C) ouabain, (D) chloroquine and (E) remdesivir for 1 h. Subsequently, the cells were incubated in the presence of indicated drug for 24 h. Statistically significantly differences between DMSO and drug treatment are represented as ****P <* 0.001. Data are presented as mean ± SD (n = 3/group). Abbreviation: ns, not significant.

**Figure S2. Progeny virus titer measurement using a plaque assay**

Progeny virus titers in the supernatant of cells following the indicated treatments for 8, 24, and 48 hpi were determined using a plaque assay.

**Figure S3. Full image of western blot**

Full image of the blots displayed in Figure 3C. Viral NP and GADPH were blotted in the same gel.
